# Volumetrie des Bulbus olfactorius – ein Baustein in der Objektivierung von Störungen des Geruchssinns

**DOI:** 10.1007/s00106-025-01650-z

**Published:** 2025-09-04

**Authors:** Stefanie Keweloh, Jörg-Michael Nebel, Daniel Overhoff, Stephan Waldeck

**Affiliations:** 1 Klinik für diagnostische und interventionelle Radiologie und Neuroradiologie, Bundeswehrzentralkrankenhaus Koblenz, Rübenacher Str. 170, 56072 Koblenz, Deutschland; 2 Klinik für Hals-Nasen-Ohrenheilkunde, Bundeswehrzentralkrankenhaus Koblenz, Koblenz, Deutschland; 3 Klinik für Radiologie und Nuklearmedizin, Universitätsmedizin Mannheim, Mannheim, Deutschland; 4 Klinik für Neuroradiologie, Universitätsmedizin Mainz, Mainz, Deutschland

**Keywords:** Riechstörung, Bulbus olfactorius, Radiologie, Magnetresonanztomographie, Olfaktometrie, Olfactory dysfunction, Olfactory bulb, Radiology, Magnetic resonance imaging, Olfactometry

## Abstract

Störungen des Geruchssinns sind ein häufiger Anlass für eine Begutachtung in der Hals-Nasen-Ohren-Heilkunde. Insbesondere bei nicht eindeutigen Ergebnissen der subjektiven und/oder objektiven Olfaktometrie bedarf es zusätzlicher objektiver Befunde, um Störungen des Geruchssinns zu verifizieren. Anhand von Erfahrungen aus der Praxis und unter Berücksichtigung der aktuellen Literatur ist dieser Artikel als eine Hilfestellung bei der Anfertigung radiologischer Zusatzgutachten zu verstehen. Dabei soll die Bedeutung einer umfassenden aktuellen Standardbildgebung einschließlich einer Bulbusvolumetrie in Zusammenschau mit einer ausführlichen Anamnese und ggf. vorhandenen Vorbefunden betont werden. Neben der Dokumentation von Veränderungen der anatomisch am Geruchssinn beteiligten Strukturen, welche die beklagten Störungen des Geruchssinns stützen, kann durch spezielle neuroradiologische Untersuchungssequenzen zur Darstellung des Bulbus olfactorius, inklusive einer volumetrischen Messung desselben, ein objektiver Bestandteil für die Beurteilung von manchmal nur subjektiv vorhandenen Störungen des Geruchssinns gewonnen werden. Da im Großteil der Fälle keine valide Vergleichsvolumetrie aus einer möglicherweise vorhandenen Vorbildgebung ableitbar ist, ist ein Vergleich mit in Studien ermittelten Vergleichswerten des Bulbus olfactorius für die jeweilige vermutete Ätiologie und das Ausmaß der mittels Olfaktometrie ermittelten/beklagten Störung die einzige Möglichkeit, das Volumen des Bulbus olfactorius in eine annähernd objektive Beziehung zur beklagten oder/und mittels Olfaktometrie ermittelten Funktion des Geruchssinns zu setzen.

## Abstract (en)

Disorders of olfaction are a frequent reason for an assessment in otorhinolaryngology. Particularly when the results of subjective and/or objective olfactometry are inconclusive, additional objective findings are required to verify disorders of the sense of smell. Based on practical experience and considering the current literature, this article is intended as an aid in the preparation of supplementary radiological reports. The importance of comprehensive up to date standard imaging including olfactory bulb volumetry in addition to a detailed medical history and, if applicable, previous findings should be emphasized. In addition to the documentation of alterations in the anatomical structures involved in olfaction, which support the reported disorders in the sense of smell, special neuroradiological examination sequences for the visualization of the olfactory bulb, including a volumetric measurement of the same, can provide an objective component for the assessment of sometimes only subjectively existing disorders of the sense of smell. In the majority of cases no valid comparative volumetry can be derived from a possibly pre-existing imaging study. Therefore, a comparison with olfactory bulb reference values, determined in studies for the presumed etiology and the extent of the disorder reported/determined by olfactometry is the only way to put the olfactory bulb volume in an almost objective relationship to the function of the sense of smell reported and/or determined by olfactometry.

Ohne Frage ist die Beurteilung von Riechstörungen immer im Zusammenhang mit dem Ereignis- respektive Unfallablauf, dem zeitlichen Verlauf der Symptomatik, ereignisnahen bzw. unfallnahen klinischen, bildgebenden und funktionsdiagnostischen Befunden sowie den gutachterlich mittels z. B. Endoskopie, subjektiver und ggf. objektiver Olfaktometrie erhobenen Befunden zu bewerten. Jedoch können die Ergebnisse von bildgebenden Verfahren in der Begutachtung bei der Objektivierung von proklamierten Riechstörungen unterschiedlicher Genese, gerade bei nicht eindeutigen Befunden in der subjektiven und objektiven Olfaktometrie, einen essenziellen Baustein darstellen. Durch die Bildgebung können mögliche zugrunde liegende strukturelle Schädigungen der am Riechsinn beteiligten neuronalen Strukturen detektiert werden. Außerdem ermöglicht sie es durch den Vergleich mit Normalbefunden, insbesondere im Rahmen einer Volumetrie des Bulbus olfactorius (BO), objektive Hinweise auf die Ausprägung von Funktionsstörungen des Riechsinns zu bekommen. Die für die Begutachtung – aus radiologischer Sicht – wichtigen Aspekte sollen im Folgenden dargestellt werden.

## Geruchssinn und mögliche Ursachen seiner Störung

Der Geruchssinn spielt in unserem Leben eine erhebliche Rolle, nicht nur weil „angenehme Gerüche“ bei der Empfindung von Genuss eine wichtige Rolle spielen, sondern auch weil „schlechte Gerüche“ uns vor der Aufnahme von möglicherweise schädlichen Stoffen schützen oder wir z. B. durch Brandgeruch vor Lebensgefahr gewarnt werden. Aber auch Berufseinschränkungen oder eine tiefgehende Verunsicherung über den eigenen Körpergeruch können die Folge von Riechstörungen sein.

Gegenüber einer Normosmie wird neben einer Anosmie und einer Hyposmie eine Vielzahl von pathologischen Veränderungen beschrieben. So vielfältig Riechstörungen sind, so vielfältig kann auch ihre Genese sein (Tab. 1). Da viele der möglichen zugrunde liegenden Erkrankungen mit Veränderungen in der Bildgebung einhergehen, ist für den radiologischen Gutachter eine genaue Anamnese unerlässlich.

**Tab. 1:** Ursachen von Riechstörungen

*Presbyosmia*
*Sinunasale Erkrankungen*
*Postinfektiös (COVID-19[„coronavirus-disease-2019“]-assoziiert, nicht COVID-19-assoziiert)*
*Posttraumatisch (inkl. Osmoagnosie [* 17*])*
*Neurologische Erkrankungen*
*Neurodegenerative Erkrankungen*
*Exposition gegenüber Drogen, Giften, Medikamenten*
*Kongenital*
*Iatrogen*
*Postradiogen*
*Nach sinunasaler oder frontobasaler Chirurgie*
*Nach Laryngektomie (durch Veränderungen des Luftflusses)*
*Tumoren und tumorähnliche Erkrankungen*
*Psychiatrische Erkrankungen*
*Systemische Erkrankungen*
*Gefäßerkrankungen*
*Idiopathisch*

## Anatomie des Geruchssinns

Voraussetzung für die Beurteilung der mithilfe bildgebender Verfahren erhobenen Befunde ist darüber hinaus eine gute Kenntnis der an der Olfaktion beteiligten anatomischen Strukturen. Abgesehen von seltenen Fällen (funktionierender Riechsinn bei radiologisch fehlenden Bulbi olfactorii (BO) [26]), erfordert der Geruchssinn ein funktionierendes Zusammenspiel zwischen der peripheren Geruchswahrnehmung durch Neurone des Riechepithels, der Signalvermittlung in den BO, der dort stattfindenden Signalintegration und schließlich der Verarbeitung in Strukturen des zentralen Nervensystems [28].

Aufgrund ihrer Bedeutung für den radiologischen Gutachter werden an dieser Stelle einzelne Aspekte herausgegriffen:

1. Axone der olfaktorischen Rezeptorneurone verlaufen zusammengeschlossen als Fila olfactoria durch die Lamina cribrosa in den BO. Schädigungen der olfaktorischen Rezeptorneurone durch normale Umwelteinflüsse werden durch die Regenerationsfähigkeit der im Riechepithel vorhandenen Stammzellen ausgeglichen.
2. Die aus dem Tractus olfactorius austretenden Stria olfactoria medialis projizieren sowohl zur ipsilateralen als auch – über die Commissura anterior – zur kontralateralen Area septalis. Über den Epithalamus stehen diese Fasern mit dem Hirnstamm in Verbindung, wodurch geruchsassoziierte Reflexe ausgelöst werden.
3. Zu den Projektionsarealen der Stria olfactoria lateralis gehören der Nucleus olfactorius anterior, der piriforme Kortex, die Amygdala und die Area entorhinalis (Brodmann-Areale 28 und 34). Sie sind im mediokaudalen Temporallappen zu finden. Zu den sekundär und tertiär involvierten Hirnarealen zählen der Hippokampus, der Parahippokampus, die Inselregion, der Gyrus cinguli und der orbitofrontale Kortex.
4. Die Areale der primären olfaktorischen Rinde, mit Ausnahme des Tuberculum olfactorium, haben rückläufige Projektionen zum BO. Der Nucleus olfactorius anterior hat sogar Verbindungen zu beiden BO [3].

## Plastizität des Bulbus olfactorius

Viele Studien zeigten, dass der BO eine Struktur mit einzigartiger Plastizität darstellt und trotz seiner interindividuellen Varianz – wie keine andere an der Olfaktion beteiligte Struktur – Hinweise auf die Funktion des olfaktorischen Systems geben kann.

Im Laufe des Lebens schwankt das physiologische Volumen des BO [12] mit einer bei Männern ab dem 40. und bei Frauen ab dem 60. Lebensjahr einsetzenden Altersinvolution [30]. Ein statistisch signifikanter Volumenunterschied des BO zwischen Rauchern und Nichtrauchern spiegelt sich allerdings nicht in einem signifikanten Unterschied im Riechvermögen wider [20]. Es konnte jedoch seit 1996 wiederholt bei zahlreichen mit Störungen der Olfaktion einhergehenden Erkrankungen eine eindeutige Korrelation zwischen der Funktionsfähigkeit des Riechsinns und dem Volumen des BO nachgewiesen werden [4, 9, 12, 18, 31]. Aus den Studien kann insbesondere abgeleitet werden, dass das Volumen des BO, abgesehen von direkten Schädigungen, sowohl durch den Informationsfluss von den Sinneszellen in der Riechschleimhaut („bottom-up effect“; [16, 28]) als auch durch Informationen aus Strukturen des zentralen Nervensystems („top-down effect“; [2, 5, 6, 14, 19, 24, 25]) beeinflusst wird. Von Mueller et al. konstatierten sogar eine Korrelation zwischen dem Ausmaß postinfektiös reduzierter Bulbusvolumina und dem Ausmaß der Störungen des Geruchssinns [18]. Laut Haehner et al. sinkt das Volumen insbesondere mit der Dauer des Verlusts des Geruchssinns [10]. Bei einer COVID-19(„coronavirus disease 2019“)-bedingten Anosmie ist ein erhöhtes Bulbusvolumen (≤ 4 Monate nach Infektion) mutmaßlich auf ein transientes inflammatorisches Ödem des BO zurückzuführen [21] und somit nicht funktionsmoduliert bedingt.

Besonders wertvoll für den Nachweis der Plastizität des Bulbusvolumens sind zudem Studien, die zeigen, dass Veränderungen des Riechvermögens eng mit Veränderungen des intraindividuellen Bulbusvolumens korrelieren [7, 8]. Intraindividuelle Seitendifferenzen des Bulbusvolumens werden in keiner Studie festgestellt. Eine Erklärung hierfür könnten die bekannten Rückprojektionen aus den Arealen der primären Riechrinde und insbesondere des Nucleus olfactorius anterior zu beiden BO sein.

Der Vollständigkeit halber ist zu erwähnen, dass beim Kallmann-Syndrom typischerweise hypoplastische oder aplastische BO gefunden werden.

## Bildgebende Verfahren

Eine umfassende Befunderhebung bildet die Basis einer objektiven Begutachtung. Grundsätzlich empfiehlt sich dazu die Zusammenschau von bereits vorhandener Bildgebung, ergänzt um ggf. eine Computertomographie (CT) und in jedem Fall eine aktuelle Magnetresonanztomographie (MRT) des Schädels. Die MRT ermöglicht die bessere Beurteilung der Weichteilstrukturen, verglichen mit der CT. Ziel einer Standardbildgebung von Hirn- und Gesichtsschädel ist neben der Erfassung intrakranieller Prozesse die Erfassung von Prozessen der Rhinobasis. Mit anderen Worten: Es sollen sinunasale Veränderungen, Gliosen, Ödeme, Defektareale, Blutungsresiduen, anatomische Varianten einschließlich der Gefäßversorgung und Tumoren detektiert werden.

**Abb. 1:**
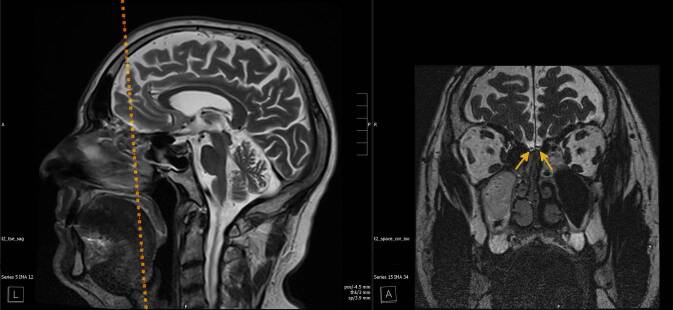
Beispielhafte Ausrichtung der koronalen Rekonstruktionen anhand einer T2-gewichteten („T2-weighted“, T2w) Turbospinecho(TSE)-Sequenz sagittal (*gestrichelte Linie*: Ebene der koronalen Rekonstruktion aus der T2w-„sampling perfection with application-optimized contrast using different flip angle evolution“[SPACE]-Sequenz [*rechts*], *Pfeile* Bulbi olfactorii)

Ergänzende, hoch aufgelöste, koronale, T2-gewichtete Sequenzen in der MRT ermöglichen darüber hinaus eine exzellente Visualisierung des BO (Abb. 1).

**Abb. 2:**
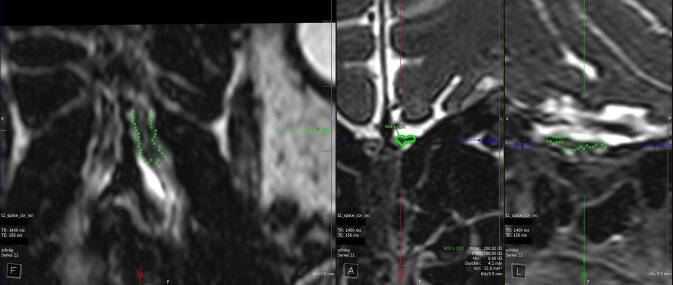
Beispielhafte Darstellung des Ergebnisses einer semiautomatischen Bulbusvolumetrie in 3 Ebenen

Wegen der besseren Auflösung empfiehlt es sich, Untersuchungen im Rahmen von Begutachtungen auf Geräten mit einer Feldstärke von 3 T durchzuführen. Neben einer Standarduntersuchung des Neurokraniums (bestehend aus T1w-Sequenzen vor und nach Kontrastmittelgabe, Diffusionsserien, suszeptibilitätsgewichteten Sequenzen, einer Time-of-Flight[ToF]-Angiographie, T2w-TSE-Sequenzen und einer „turbo inversion recovery magnitude“[TIRM]-Dark-fluid-Sequenz) ist die ergänzende Durchführung einer isotropen, hoch aufgelösten, koronalen, T2w-TSE/„fast spin echo“(FSE)-Sequenz über die vordere und die mittlere Schädelbasis in Nativtechnik opportun. Die hier akquirierten isotropen Bilder sollten um Rekonstruktionen in koronaler Ausrichtung mit einer Schichtdicke von 0,5 mm ergänzt werden (technische Details siehe Infobox). Diese Rekonstruktionen sollten schließlich für eine Volumetrie des BO (rechts und links getrennt) verwendet werden. Dies kann z. B. mittels semiautomatischer Volumenerfassung durch manuelles Umfahren der Konturen erfolgen (Abb. 2). Das proximale Ende des BO wird durch einen Kalibersprung am Beginn des Tractus olfactorius definiert. Das Ergebnis der Berechnung sollte in Kubikmillimetern (mm^3^), getrennt für den rechten und den linken BO, dokumentiert werden. Das Gesamtvolumen setzt sich aus der Summe der ermittelten Einzelwerte zusammen.

### Infobox

Konkretes technisches Beispiel für eine isotrope, hoch aufgelöste, koronale, T2-gewichtete Sequenz (3-D[dreidimensionale]-TSE-Sequenz)

- Hersteller: Siemens AG, Erlangen, Deutschland
- Modell: Magnetom Vida fit
- Feldstärke: 3 Tesla (3 T)
- Untersuchungssequenz: T2-SPACE(„sampling perfection with application-optimized contrast using different flip angle evolution“)-Sequenz in koronaler Ausrichtung (isotropic [iso] koronal)
- Schichtdicke: 0,5 mm
- Repetitionszeit („time of repetition“, TR): 1400 ms
- Echozeit („time to echo“, TE): 156 ms
- Turbofaktor („echo train length“, ETL): 69
- Flipwinkel („Flip angle“): 120
- „Field of view“ (FOV): 180 × 180 mm^2^
- Lokalisation: über die vordere und mittlere Schädelbasis

Der Vollständigkeit halber soll an dieser Stelle erwähnt werden, dass entsprechende dreidimensionale (3-D) TSE/FSE-Sequenzen im Portfolio anderer MRT-Geräte-Hersteller zu finden sind. General Electric (GE) Healthcare verwendet die Bezeichnung CUBE, Philips die Bezeichnung VISTA („volume isotropic turbo spin echo aquisition“), bei Canon heißt die Sequenz 3D Multivoxel (MVOX), und bei Hitachi heißt sie isoFSE.

## Alternative Ansätze in der Bildgebung

Aufgrund der fehlenden validen Datenlage bei Patienten mit posttraumatisch bedingten Störungen des Geruchssinns, der sich auf Forschungsniveau bewegenden Komplexität einzelner Untersuchungsverfahren, der aktuell fehlenden Relevanz im gutachterlichen Setting sowie der fehlenden Validität der Datenakquise finden einige vielversprechende Untersuchungsansätze aktuell keine Anwendung im Rahmen der Begutachtung. Dennoch sollen sie an dieser Stelle zumindest erwähnt werden:

- Messungen der Tiefe des Sulcus olfactorius [13, 23],
- Aktivitätsnachweis in verschiedenen olfaktorischen Kortexarealen im Rahmen einer funktionellen MRT [27],
- Volumetrie einzelner Kortexareale bei Störungen des Geruchssinns [11],
- Diffusions-Tensor-Bildgebung („diffusion tensor imaging“, DTI) des BO bei COVID-19-assozierter Anosmie [21],
- Signalintensitätsmessungen des BO [22].

## Begutachtung

Häufig sind berufsgenossenschaftlich versicherte oder unfallversicherte posttraumatische Störungen des Geruchssinns Anlass für eine Begutachtung. Verletzungen der ossären Strukturen im Bereich der Lamina cribrosa in einer CT legen einen Zusammenhang mit beklagten Riechstörungen, z. B. im Rahmen eines (Teil‑)Abrisses der Fila olfactoria, nahe. Blutungsresiduen sowie Gliosen im Bereich des Kortex, insbesondere im Frontal- und im anteroinferioren Temporallappen, sind z. B. ein starker Hinweis darauf. Aber auch indirekte Schäden durch ein Ödem sind zu berücksichtigen [15]. Neben Monoverletzungen kann es parallel aber auch zu Verletzungen auf dem gesamten Signalweg der Geruchsinformationen und -verarbeitung gekommen sein, d. h., neben Abscherverletzungen der Axone der Rezeptorneurone können sowohl der BO als auch nachgeschaltete Strukturen des zentralen Nervensystems direkt geschädigt worden sein.

Leider sind mutmaßlich zugrunde liegende Ursachen nicht immer so offensichtlich. Bei Begutachtungen werden häufig Riechstörungen untersucht, die nicht mit eindeutigen pathologischen Veränderungen anatomischer Strukturen in der Standardbildgebung erfasst und klinisch vielleicht auch nicht eindeutig im Rahmen einer Olfaktometrie belegt werden können.

Hier unterscheidet sich die Bildgebung im klinischen Kontext, wo sie angepasst an die vermutete zugrunde liegende Ätiologie der Störung des Geruchssinns erfolgt, von der im Gutachtenwesen. Im Kontext der Begutachtung verfolgt die Bildgebung einen allumfassenden Ansatz zur Erfassung einer möglichen Ätiologie und die Anwendung objektiver Verfahren zur Verifikation. Neuroradiologische Spezialuntersuchungen und Messmethoden werden somit zu einem Baustein in der Objektivierung der beklagten Beschwerden. Bei ihrer Auswahl ist auf eine wissenschaftliche Belastbarkeit der Verfahren zu achten.

Die Volumetrie des BO ist, geschlechts- und alterskorreliert, ein objektives Werkzeug zur Definition eines normalen, hypoplastischen oder aplastischen BO. Unter Zuhilfenahme der 10. Perzentile einer Verteilungskurve setzen Buschhüter et al. ein pathologisches Bulbusvolumen für Männer und Frauen im Alter unter 45 Jahren mit einem Wert kleiner als 58 mm^3^ und für Männer und Frauen über 45 Jahre mit einem Wert kleiner als 46 mm^3^ [4] an. Noch genauer haben Bauknecht et al. aufgrund der guten Korrelation zwischen objektiver Olfaktometrie und der Volumetrie des BO folgende Werte des Gesamtbulbusvolumens festgestellt:

- Probanden ohne Riechstörung: 110,7 mm^3^ ± 21,5 mm^3^;
- Personen mit einer Hyposmie: 50,5 mm^3^ ± 25,5 mm^3^;
- Personen mit einer Anosmie: 15,7 mm^3^ ± 23,3 mm^3^).

Die zugehörige ROC(„receiver operating characteristic“)-Analyse ergab Cut-off-Werte von 32 mm^3^ für eine Anosmie und von 80,7 mm^3^ für eine Hyposmie [1]. Die bei Mueller et al. recht hohen Normwerte gesunder Probanden für das Bulbusvolumen beidseits (rechts: 93,5 mm^3^, links 91,6 mm^3^) bei gleichzeitig großen interindividuellen Schwankungen sind am ehesten der verwendeten niedrigeren Feldstärke gegenüber späteren Untersuchungen geschuldet. Nichtsdestotrotz konnte von ihnen eine signifikante Reduktion der Bulbusvolumina bei Patienten mit posttraumatischen und postinfektiösen Störungen des Geruchssinns nachgewiesen werden [18]. Ergänzend zur Volumetrie des BO, empfiehlt sich auf Basis einer noch sehr jungen Studie von Yan et al., die Form der BO zu betrachten. Bei posttraumatisch bedingten Störungen des Geruchssinns detektierten sie signifikant häufiger aufgefaserte/geteilte Bulbi [29].

### Anmerkung.

Eine Bulbusvolumenreduktion oder eine ausgeprägte Seitendifferenz der Bulbusvolumina bis hin zum fehlenden Nachweis eines oder beider BO kann im Falle einer Traumaanamnese Ausdruck einer schweren direkten Schädigung sein. Jedoch kann sie auch unter Hinzunahme möglicherweise vorliegender Voraufnahmen zumeist nicht von einer möglichen kongenitalen Bulbushypoplasie oder gar Bulbusaplasie differenziert werden.

## Fall aus der Praxis

**Abb. 3:**
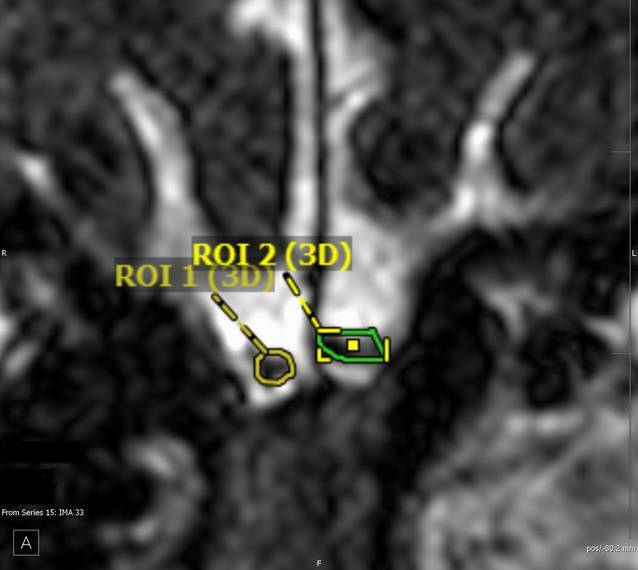
Kontur der Bulbi olfactorii nach manuellem Umfahren in koronaler Ebene im Rahmen der Volumenmessung

Abb. 3 zeigt einen Fall aus der Praxis.

### Anamnese.

Seit mehreren Jahren bestehende Riechstörung nach einem Arbeitsunfall mit Schädel-Hirn-Trauma, Begutachtung im Rahmen eines Versicherungsfalls.

### Voruntersuchungen.

Mehrere ≥ 1,5 Jahre alte CT- und MRT-Untersuchungen ohne hoch auflösende Darstellung der Frontobasis des Neurokraniums.

### Bildgebung.

Umfassende Standard-3T-MRT, ergänzt um eine dünnschichtige 3D-TSE-Sequenz in koronaler Ausrichtung über der Frontobasis.

### Pathologische Befunde in der 3T-MRT.

Floride sinusitische Veränderungen in den Nasennebenhöhlen, ein chronisch-entzündlicher Prozess im rechten Felsenbein, unspezifisch mikroangiopathische Marklagerveränderungen und kleinfokale, a. e. posttraumatische Glioseareale rechts temporopolar sowie juxta-/subkortikal und beidseits hochfrontal.

### Ergebnis der Volumetrie des BO.

Rechts: 39 mm^3^, links: 50 mm^3^

### Beurteilung.

Die reduzierten Bulbusvolumina stützen in Zusammenschau mit den diskreten, a. e. posttraumatischen gliotischen Veränderungen der an der Olfaktion beteiligten Strukturen die vom Versicherten geschilderten Beschwerden. Ein durch Scherkräfte bedingter (Teil‑)Abriss der Fila olfactoria wäre aufgrund des Traumamechanismus eine mögliche und auch naheliegende Erklärung für die in der MRT erhebbare Bulbushypotrophie.

## Fazit für die Praxis

- Es empfiehlt sich, in der Begutachtung eine 3T-Magnetresonanztomographie (MRT) des Neurokraniums inkl. Volumetrie der Bulbi olfactorii (BO) durchzuführen.
- Die Volumetrie des BO ist eine zuverlässige und objektive Methode, um die Funktion des Riechsinns zu evaluieren.
- Die ermittelten Volumina für die BO sollten erstens mit dem typischerweise bei Personen mit einer durch eine objektive Olfaktometrie festgestellten und entsprechend ausgeprägten Riechstörung ermittelten Volumenwertebereich des BO verglichen werden. Zweitens sollten sie mit dem typischerweise bei Personen mit einer ähnlichen Ätiologie der Riechstörung ermittelten Volumenwertebereich des BO verglichen werden. Auf diese Weise kann eine beklagte Hyposmie/Anosmie bei grenzwertigem Normalbefund der Bulbusvolumetrie, d. h., wie er typischerweise bei einer Normosmie gefunden wird, teilweise doch noch aufgrund der Ätiologie mit einem ätiologietypischen pathologischen Bulbusvolumen korreliert werden.
- Die Funktionalität der Gesamtheit der unterschiedlichen am Geruchssinn beteiligten Strukturen fungiert aufgrund des Bottom-up- und des Top-down-Effekts als Modulator des Bulbusvolumens.
- Die gutachterliche Stellungnahme sollte schließlich darauf eingehen, ob ein Anhalt für andere mögliche Ursachen für die vom Versicherten geschilderten Beschwerden zu finden sind.
